# Noninvasive optoacoustic imaging of breast tumor microvasculature in response to radiotherapy

**DOI:** 10.3389/fphys.2022.1044308

**Published:** 2022-10-17

**Authors:** Dan Wu, Nan Xu, Yonghua Xie, Yang Shen, Yunlu Fu, Liang Liu, Zihui Chi, Runyu Lu, Renjie Xiang, Yanting Wen, Jun Yang, Huabei Jiang

**Affiliations:** ^1^ School of Optoelectric Engineering, Chongqing University of Posts and Telecommunications, Chongqing, China; ^2^ Department of Radiology, The Third Affiliated Hospital of Kunming Medical University, Yunnan Cancer Hospital, Kunming, China; ^3^ Ultrasonic Department, The Fifth People’s Hospital of Chengdu, Chengdu, China; ^4^ Department of Medical Engineering, University of South Florida, Tampa, FL, United States

**Keywords:** optoacoustic imaging, radiotherapy, breast tumor, tumor microvasculature, oxygenation status

## Abstract

Detailed insight into the radiation-induced changes in tumor microvasculature is crucial to maximize the efficacy of radiotherapy against breast cancer. Recent advances in imaging have enabled precise targeting of solid lesions. However, intratumoral heterogeneity makes treatment planning and monitoring more challenging. Conventional imaging cannot provide high-resolution observation and longitudinal monitoring of large-scale microvascular in response to radiotherapy directly in deep tissues. Herein, we report on an emerging non-invasive imaging assessment method of morphological and functional tumor microvasculature responses with high spatio-temporal resolution by means of optoacoustic imaging (OAI). *In vivo* imaging of 4T1 breast tumor response to a conventional fractionated radiotherapy at varying dose (14 × 2 Gy and 3 × 8 Gy) has been performed after 2 weeks following treatment. Remarkably, optoacoustic images can generate richful contrast for the tumor microvascular architecture. Besides, the functional status of tumor microvasculature and tumor oxygenation levels were further estimated using OAI. The results revealed the differential (size-dependent) nature of vascular responses to radiation treatments at varying doses. The vessels exhibited an decrease in their density accompanied by a decline in the number of vascular segments following irradiation, compared to the control group. The measurements further revealed an increase of tumor oxygenation levels for 14 × 2 Gy and 3 × 8 Gy irradiations. Our results suggest that OAI could be used to assess the response to radiotherapy based on changes in the functional and morphological status of tumor microvasculature, which are closely linked to the intratumor microenvironment. OAI assessment of the tumor microenvironment such as oxygenation status has the potential to be applied to precise radiotherapy strategy.

## 1 Introduction

Tumor microenvironment, such as oxygenation state, has a great influence on the radiosensitivity of tumor cells. Tumor microenvironment is closely related to the function of tumor microvessels. Therefore, a detailed understanding of radiation-induced changes in tumor microvessels is essential to maximize the efficacy of radiation therapy for cancer (Park et al., 2012). The main manifestations of tumor microvessels response to irradiation are decreased perfusion, increased vascular wall permeability, decreased number of functional vessels, decreased vascular network density, vascular contraction, and decreased blood flow velocity (Pedro et al., 2019; Anamitra et al., 2022). Research has found that the therapeutic ratio in treating cancer with radiation could be increased by delivering the radiation in multiple fractions, fractionated radiotherapy has been an almost universally accepted clinical practice (Brand Douglas et al., 2019). The effect of radiation on tumor microvessels varies with the changes of total dose, dose rate, dose fraction and fraction number, as well as biological factors such as tumor type, tumor site and tumor growth stage (Yoon Stephanie et al., 2021).

Studying the mechanisms of tumor tissue destruction by different doses of radiation, especially its microvascular response, is crucial for optimizing treatment plan, prognosis and follow-up. Noninvasive imaging methods offer the potential for longitudinal monitoring of dynamic temporal changes occurring in the tumor microenvironment and allow us to map the spatial heterogeneity of tumor microvessels and tumor oxygenation. Conventional imaging methods such as magnetic resonance imaging (MRI) (Morgan Tiffany et al., 2019; Turkkan et al., 2022), positron emission tomography (PET) (Abravan et al., 2017; Jiang et al., 2021) and computed tomography (CT) (Wang et al., 2011; Crane Christopher and Koay Eugene, 2016) have been used to study tumor blood vessels and oxygenation in animal models and patients. However, these technologies are either more expensive or require the use of ionizing radiation or radioactive isotopes. The penetration depth of Doppler ultrasound imaging (UI) is appropriate, but the contrast and spatial resolution are insufficient to clearly identify slow blood flow microvessels (McNabb et al., 2020). Therefore, it is of great clinical value to study a cheap, reliable and simple method of tumor microvessels and oxygenation imaging.

Optoacoustic imaging (OAI) is a hybrid imaging technology that combines the sensitivity of optical imaging with the resolution of ultrasonic imaging (Jiang, 2014; Beard Paul and Cox Ben, 2016; Vasilis and Buehler, 2017; Liu et al., 2019). In OAI, tissues are irradiated with near-infrared light, which is absorbed by endogenous chromophores such as oxygenated and deoxygenated hemoglobin, causing thermoelastic expansion and producing broad-band pressure waves that are detected as acoustic signals (Wang, 2016; Duan et al., 2021; Wang et al., 2021; Gong et al., 2022). Interestingly, the absorption properties of hemoglobin are affected by whether or not it binds to oxygen. By comparing the PA signals of deoxyhemoglobin (Hb), oxyhemoglobin (HbO) and oxygen saturation (sO_2_) could be calculated in intratumoral and peritumoral areas (Huang et al., 2016; Rich Laurie and Mukund, 2016; Dai et al., 2020; Jiao et al., 2020; Qian et al., 2020). In this way, OAI uses endogenous contrast mechanisms to visualize tumor microvascular structure and hemodynamics. As a result, there is widespread interest in developing OAI for medical applications, particularly in cancer research.

In this study, we used the OAI system based on a semicircular array detector to monitor the response of tumor vascular networks to multiple exposures to different doses of ionizing radiation. It meets our requirements for real-time monitoring and quantitative analysis of tumor vessels, especially to improve the imaging sensitivity of microvessels. Using the xenograft mouse model of 4T1 breast cancer, we demonstrated the differential (size dependent) nature of *in vivo* microvascular response to radiotherapy. Multispectral measurements were further used to monitor tumor hemodynamic parameters.

## 2 Methods and materials

### 2.1 *In vivo* models and cell lines

The experiments were carried out on nine 5-6-week-old female Balb/c mice (18–20 g). The animals were obtained from the department of experimental animals, Kunming Medical University (Kunming, China). The study was performed on the model of murine breast cancer cells 4T1 (Chinese Academy of Sciences Stem Cell Bank). The cells were cultured in RPMI-1640 containing 10% fetal bovine serum and penicillin-streptomycin. 1 × 10^6^ cells in 200 μL of PBS were injected subcutaneously into the outer side of the right lower limb. The animal studies were approved by the Ethics Committee of Chongqing University of Posts and Telecommunications and Kunming Medical University.

### 2.2 Irradiation treatment and experimental protocols

**FIGURE 1:**
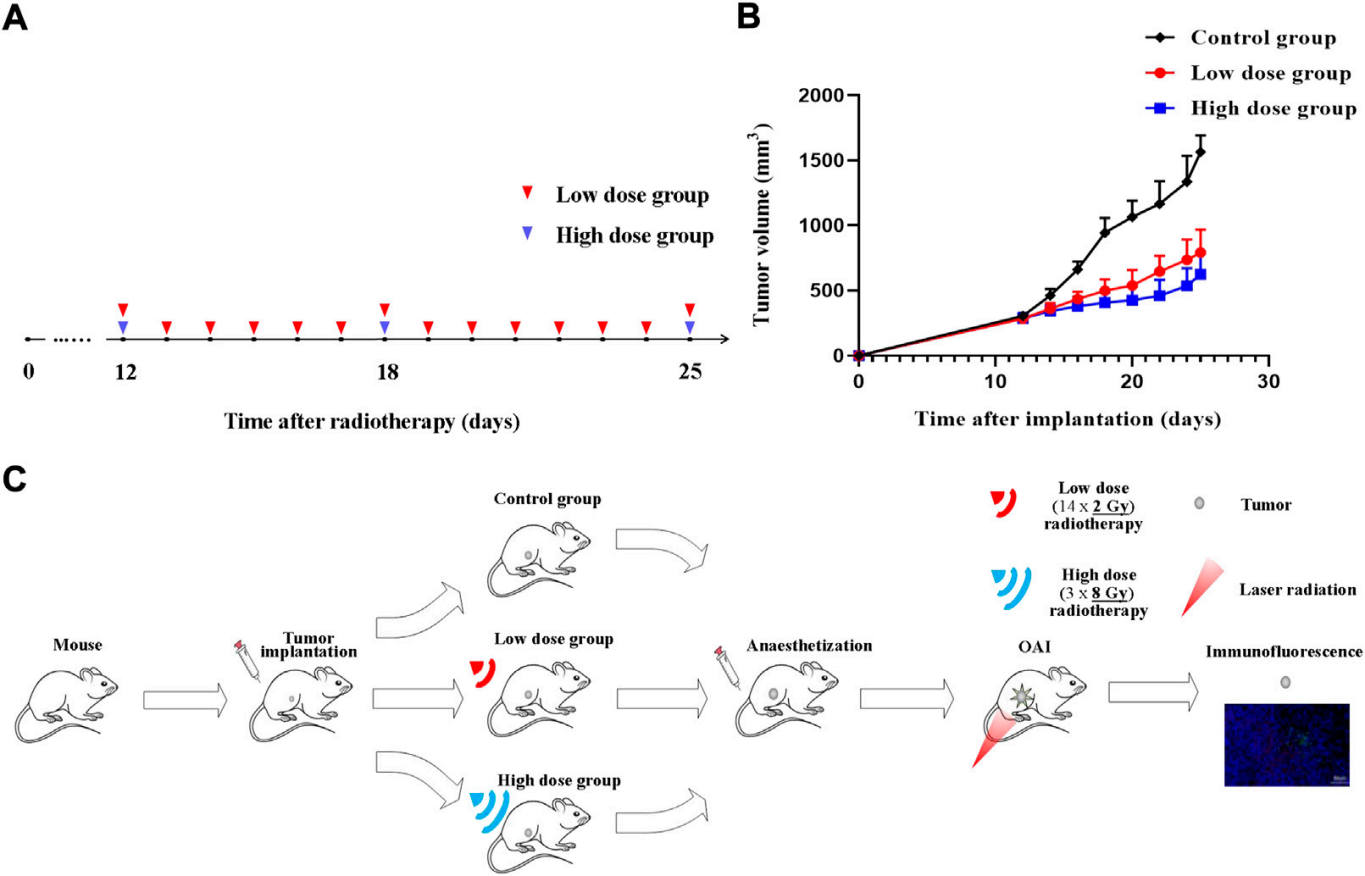
Radiotherapy protocol during tumor growth process. Experiment schedule **(A)** and dynamics of 4T1 volume after single-dose irradiation at different doses **(B,C)** Illustrative overview of experimental protocols. Time point of tumors radiotherapy is indicated by blue triangle (high dose) and red triangle (low dose).

Irradiation was performed with RS2000 Biological X-ray Biological Irradiator (VGeorgia, United States) in the 6X SRS mode with an accelerating voltage of 225 kV and a dose rate of 1.8 Gy/min. The mice were immobilized so that an irradiation field of 8 × 8 m^2^ was formed at the tumor and the rest of the body was covered with a lead screen to reduce radiation to the normal tissue. The tumor-bearing mice were randomly divided into three groups: low dose radiation group, high dose radiation group and control group. Animals (three mice in a group) received 14 × 2 Gy (low dose) or 3 × 8 Gy (high dose) radiation for two consecutive weeks (Figure 1A). Three mice were used as an untreated control. Tumor volume and body weight were measured every 2 days. Tumors were measured along two perpendicular directions with a caliper, and their volumes were calculated as follows: V = 0.5 × a × b^2^, where V is the volume and a and b are the two corresponding diameters (Figure 1B).

OA imaging was performed after tumor fractionated radiotherapy. Mice were anesthetized with chloral hydrate by 10% chloral hydrate solution which was injected intraperitoneally to the animal with a dose of 4 ml/kg body weight in all the experiments. After OA investigation, all mice were sacrificed by spinal dislocation. The tumor tissues were collected for immunofluorescence staining. Figure 1C shows an illustrative overview of experimental protocols.

### 2.3 Optoacoustic imaging system and data processing

**FIGURE 2:**
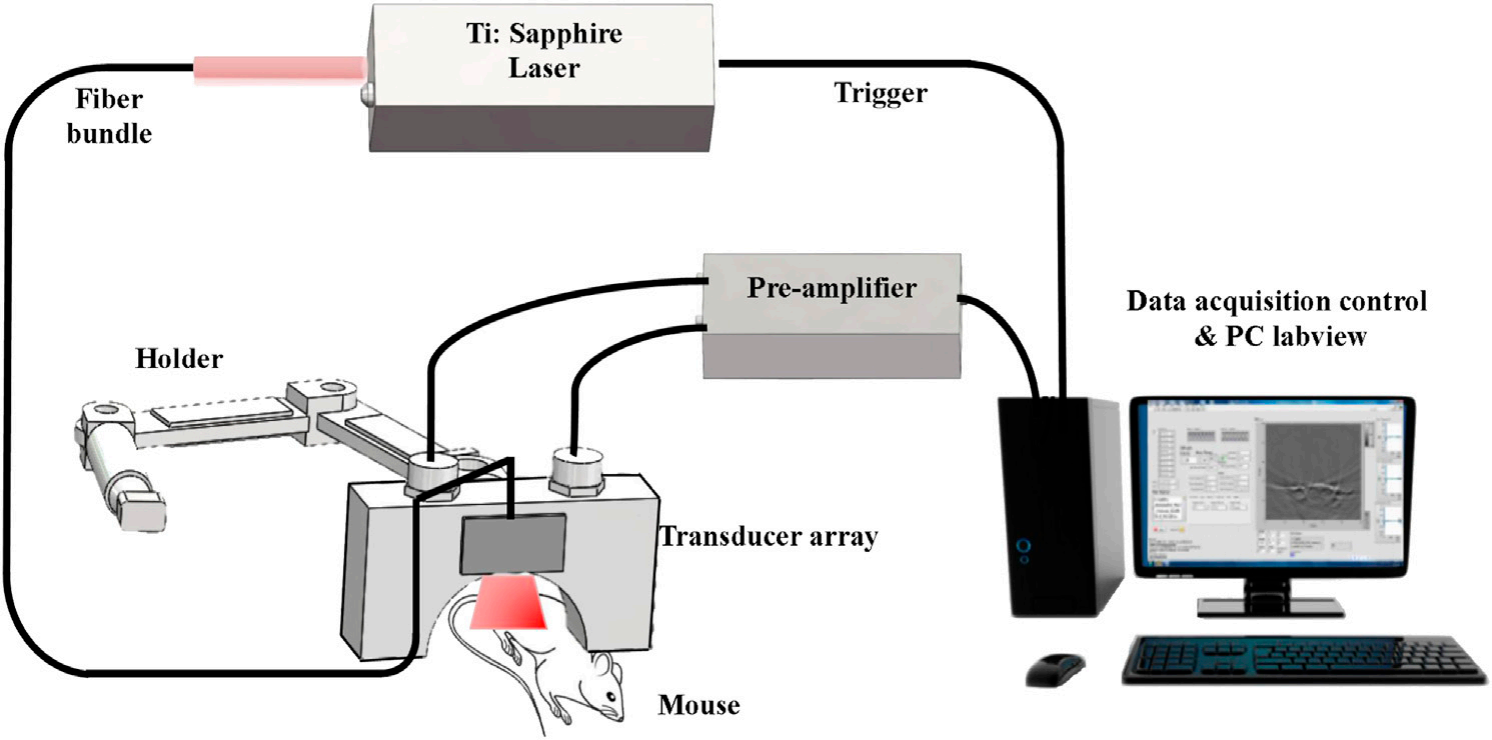
Schematic of our OAI system for *in vivo* imaging tumor-bearing mouse.

An *in vivo* laboratory-built OAI system was used in this study. The schematic diagram of OAI system for monitoring the effects of radiotherapy on tumor microvessels in this study is shown in Figure 2. In this system, a pulsed Ti:Sapphire laser (Surelite OPO, Continuum United States) with 6–7 ns pulsed duration, 20 Hz pulse repetition rate and wavelengths of 690–960 nm was used as the excitation source. The maximum fluence at 690–960 nm was approximately 18 mJ/cm^2^ during the experimentation. A custom-made fiber bundle with line-shaped illumination pattern (40 × 1 mm^2^) was applied to deliver the light from a pulsed laser. The energy of pulsed laser was controlled below 8 mJ/cm^2^ at the surface of the animal’s skin.

A 128 element ultrasound transducer array (center frequency 5 MHz, bandwidth 90%, Japan Probe Co., Ltd.) to receive the photoacoustic signal. The received photoacoustic signal is amplified by a home-made preamplifier (gain 54 dB, frequency range: 200 kHz-15 MHz), and the amplified OA signals were then collected by 128-channel data acquisition cards at a sampling rate of 50 MS/s and 12-bit digital resolution (PXIe5105, National Instrument, United States). Ten-time averaging of the signal can minimize the unstability of laser energy, and thus the image quality is improved, making the calculation of tumor area more accurate. One complete frame of data from single wavelength was acquired in 0.5 s. During the acquisition process, real-time imaging can be realized on the Labview panel. The OAI tumor images were reconstructed from the photoacoustic signals using a multispectral quantitative reconstruction algorithm. The in-plane spatial resolution of the system is about approximately 150 μm according to our previous research (Yang et al., 2020a).

PAT tumor images were reconstructed from photoacoustic signals using a delay and sum reconstruction algorithm. According to the algorithm in our previous article (Yang et al., 2020a; Yang et al., 2020b), we can obtain the tumor deoxyhemoglobin (Hb), oxyhemoglobin (HbO), oxygen saturation (sO_2_) and water (H_2_O) changes of tumor regions at three different wavelengths (760 nm, 840 nm, and 930 nm).

In each OA parameter image, an area of 20 × 20 pixels on the normal tissue surrounding the tumor, the boundary of the tumor and internal tumor was selected as three regions of interest (ROI I, ROI II, and ROI III). The mean pixel value of the ROIs was calculated. The ROI I was used as a baseline to calculate the relative changes on ROI II and ROI III. The resulting OA images were processed by background elimination and normalization. In the method of background elimination, the OA signals below the baseline were partly removed, and only the rest of the signals were retained (incomplete background elimination). This method can improve the signal-to-noise ratio. Furthermore, to conveniently calculate the pixel value within the ROIs, regions outside the ROIs were assigned a pixel value of zero. We can calculate the pixel value according to the following formula: SUM = J × P, where P and J are the pixel matrix of the reconstructed image and the binary image matrix, respectively. First, the template image of the regions of interest (ROIs) is transformed into the binary image matrix J, where regions outside the ROIs were assigned a pixel value of zero. We then obtained the pixel value, that is, the normalized optical contrast value, by multiplying J and P. Finally, the pixel values for each ROI were calculated.

### 2.4 Tumor immunofluorescence

Tumor tissues were fixed with 4% paraformaldehyde and cut into 4-μm-thick sections after dehydration and embedment. In brief, specimens were incubated with anti-CD31 (1:500, Abcam, ab182981), anti-α-SMA (1:200, Servicebio, GB13044) antibodies for staining. The slices were imaged with a NIKON ECLIPSE C1 fluorescence microscope and scanned and analyzed with a PANNORAMIC panoramic slice scanner and Image-Pro Plus 6.0 analysis software. Tumor slices were divided into three equal areas, and the microvessel density (MVD) of each area was detected by CD31 staining of perivascular cells, and the area was quantified on the basis of the total number of microvessels per unit area; similarly, the coverage of the perivascular cells stained for α-SMA in each area was calculated. The vascular maturity index (VMI) refers to the percentage of blood vessels stained with the anti-α-SMA antibody compared to the total number of blood vessels stained with CD31.

### 2.5 Statistical analysis

All measurement data obtained for tumor growth are shown as mean ± SD; deoxyhemoglobin (Hb), oxyhemoglobin (HbO) and oxygen saturation (sO_2_) are shown as means ± SEM. Parameters of microvessel density (MVD), microvessel segments number, and size are shown as the line chat including means, minimum and maximum values of the data set. SPSS 25.0 software (SPSS Company, Chicago, Illinois, United States) was used for statistical analysis. Statistically significant value was taken as *p* ≤ 0.05.

## 3 Results

### 3.1 Tumor growth after irradiation

The volume in control group increased from 306.38 ± 21.33 mm^3^ (on day 12) to 1,566.37 ± 125.91 mm^3^ (on day 25). Irradiated tumors appeared to be of smaller size as compared to untreated ones at the time of monitoring completion. Differences between treated and untreated tumors on 20th day of the experiment were assessed for all groups. Tumor growth inhibition index (TGI) were calculated as follows: TGI = 1-Relative Tumor Volume (irradiation group)/Relative Tumor Volume (control group). TGI was 49.28% for 14 × 2 Gy-irradiated tumors and 60.01% for tumors irradiated with 3 × 8 Gy. Compared with the control group, radiotherapy significantly inhibited the tumor progression. The tumor growth in the high dose group was lower than that in the low dose group (Figure 1B).

### 3.2 Optoacoustic imaging

**FIGURE 3:**
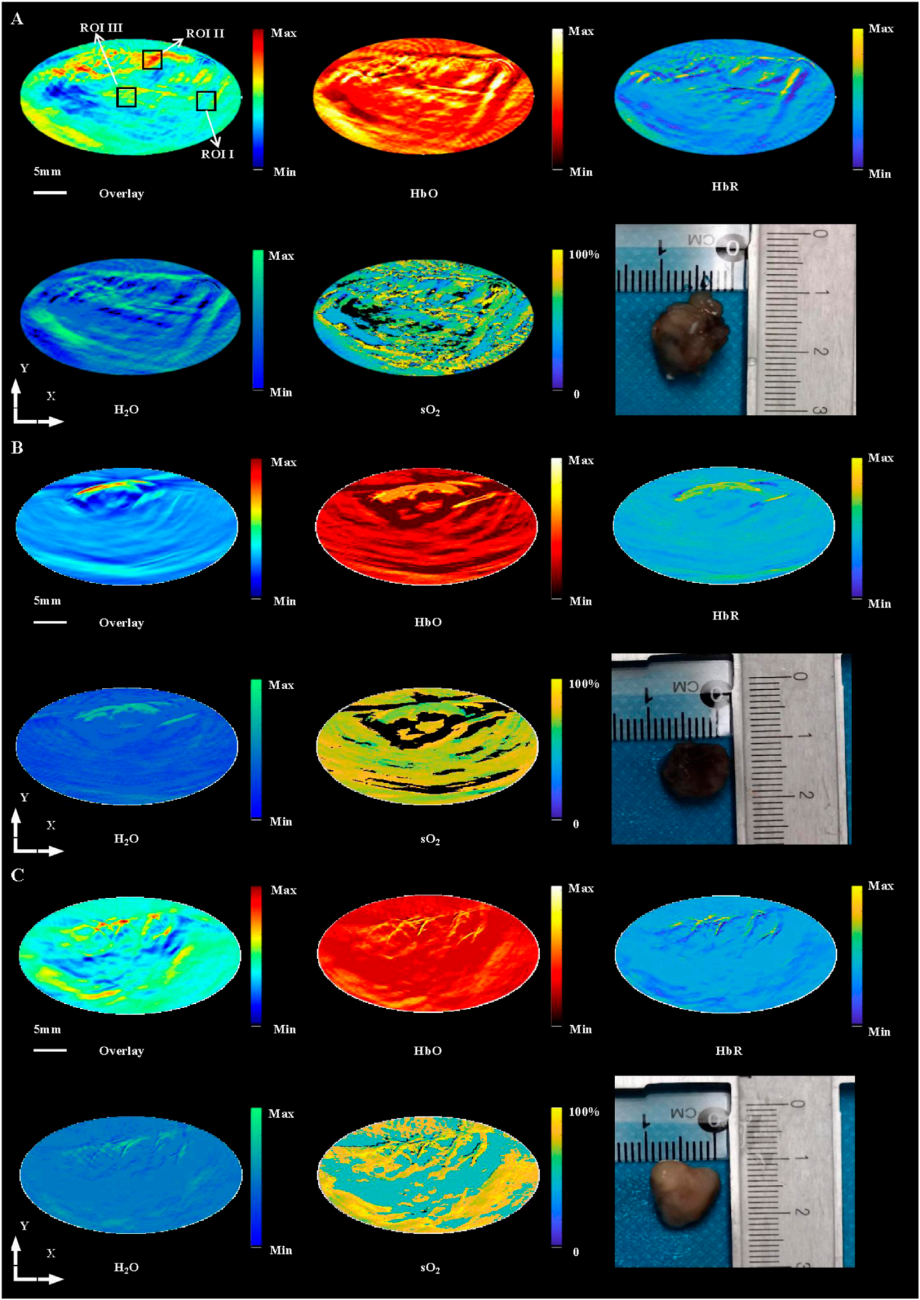
*In vivo* imaging of 4T1 breast tumors at HbT (overlay), HbO, HbR, H_2_O and sO_2_ distribution in control group **(A)**, 14 × 2 Gy group **(B)** and 3 × 8 Gy group **(C)** at 25 days after radiotherapy, and histological photograph of three collected tumor tissues in different groups. ROI I, ROI II, and ROI III in (A) indicates the baseline, peritumor and intratumor respectively. Scale bar = 5 mm for the images.

**FIGURE 4:**
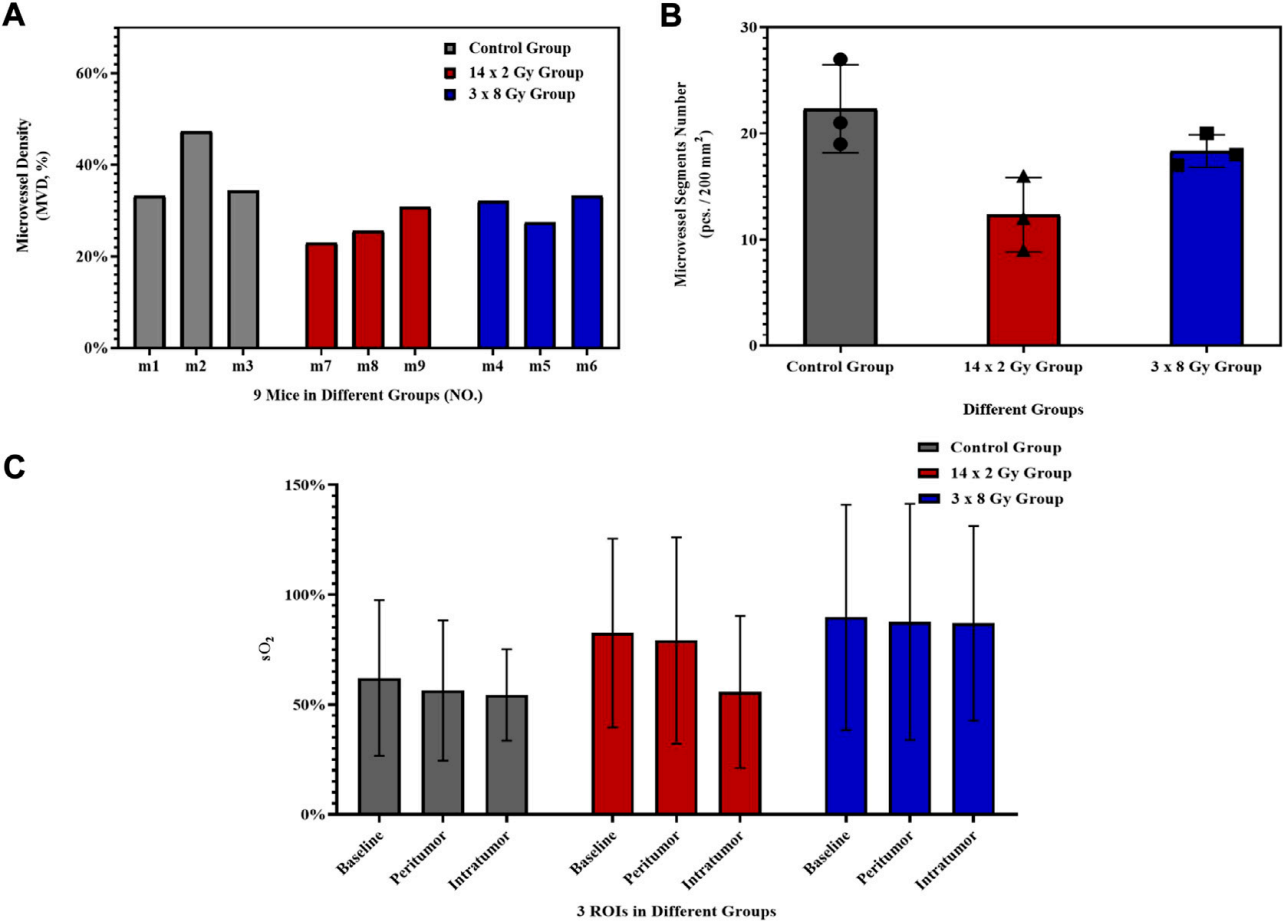
Histogram of OA parameters. Histogram of the percentage variation (SEM) of microvessel density (MVD, 100%) **(A)** and microvessel segments number **(B)** of 4T1 breast tumors after irradiation at different doses. **(C)** Histogram of the percentage variation (SEM) of sO_2_ at baseline, peritumor and intratumor at different doses. The error bar indicated the standard deviation.

Using OAI system, nine mice in control group (Figure 3A), 14 × 2 Gy group (Figure 3B) and 3 × 8 Gy group (Figure 3C) were scanned at 25 days after radiotherapy. One representative mouse was selected in each group. HbT (an overlay of 760 nm image, 840 nm image and 930 nm image), HbO, HbR, H_2_O, and sO_2_ distribution images were shown in Figure 3. As evidenced by the OA images, the tumors presented as regions containing small, broken, irregular, randomly distributed blood vessels that were markedly different from the normal microvessels in the surrounding healthy tissue. Untreated 4T1 breast tumor was characterized by a more extensive distribution of hemoglobin in the tumor peripheral area and inside the tumor. Elongated small vascular structures formed from the inside to the surface of the tumor, followed by superficial scabs at later stages of tumor development. The sO_2_ distribution on the surface and inside the tumor was relatively uniform. Histological photographs of three collected tumor tissues in different groups indicated that the tumor size in the control group was larger than that in the irradiation group. After irradiation, only short fragments of blood vessels retained in the tumor area, and the tumor microvessel density and the water content was decreased overall. Compared with the control group, the hemoglobin concentration at the tumor boundary decreased slightly, while the hemoglobin concentration inside the tumor decreased significantly in the irradiation group. After irradiation, the internal sO_2_ distribution is very uneven. The response was more pronounced at 14 × 2 Gy irradiation dose than at 3 × 8 Gy irradiation dose. OA data of all tumors in different groups were statistically analyzed. Microvessel density (MVD, 100%) was determined as a percentage of tumor area occupied by microvessels. Number of microvessel segments was calculated as a ratio between the number of microvessel fragments and the corresponding vessel area. MVD, microvessel segments number and sO_2_ were used to evaluate the effect of fractionated radiotherapy at varying dose on tumor angiogenesis. Compared with the control group, the number of blood vessels decreased, the 14 × 2 Gy group decreased to 30.89%, the 3 × 8 Gy group decreased to 19.18%. There was a significant difference in MVD between the irradiation group and the control group (Figure 4A). In addition, compared with the control group, radiotherapy decreased the number of microvessel segments, the 14 × 2 Gy group decreased by 44.78%, the 3 × 8 Gy group decreased by 17.91%, there was a more significant difference in microvessel segments number in the 14 × 2 Gy group and the increase in 3 × 8 Gy group was not statistically significant (Figure 4B). Finally, the percentage variation (SEM) of sO_2_ at ROI I, ROI II and ROI III at different doses were statistically analyzed. ROI I, ROI II and ROI III in Figure 3A represents the baseline, peritumor and intratumor respectively. Compared with the control group, sO_2_ in irradiation group increased remarkably. There was a significant difference in sO_2_ at intratumor between 3 × 8 Gy group and the control group. Besides, there was a significant difference in sO_2_ at baseline and peritumor between the irradiation group and the control group (Figure 4C).

**FIGURE 5:**
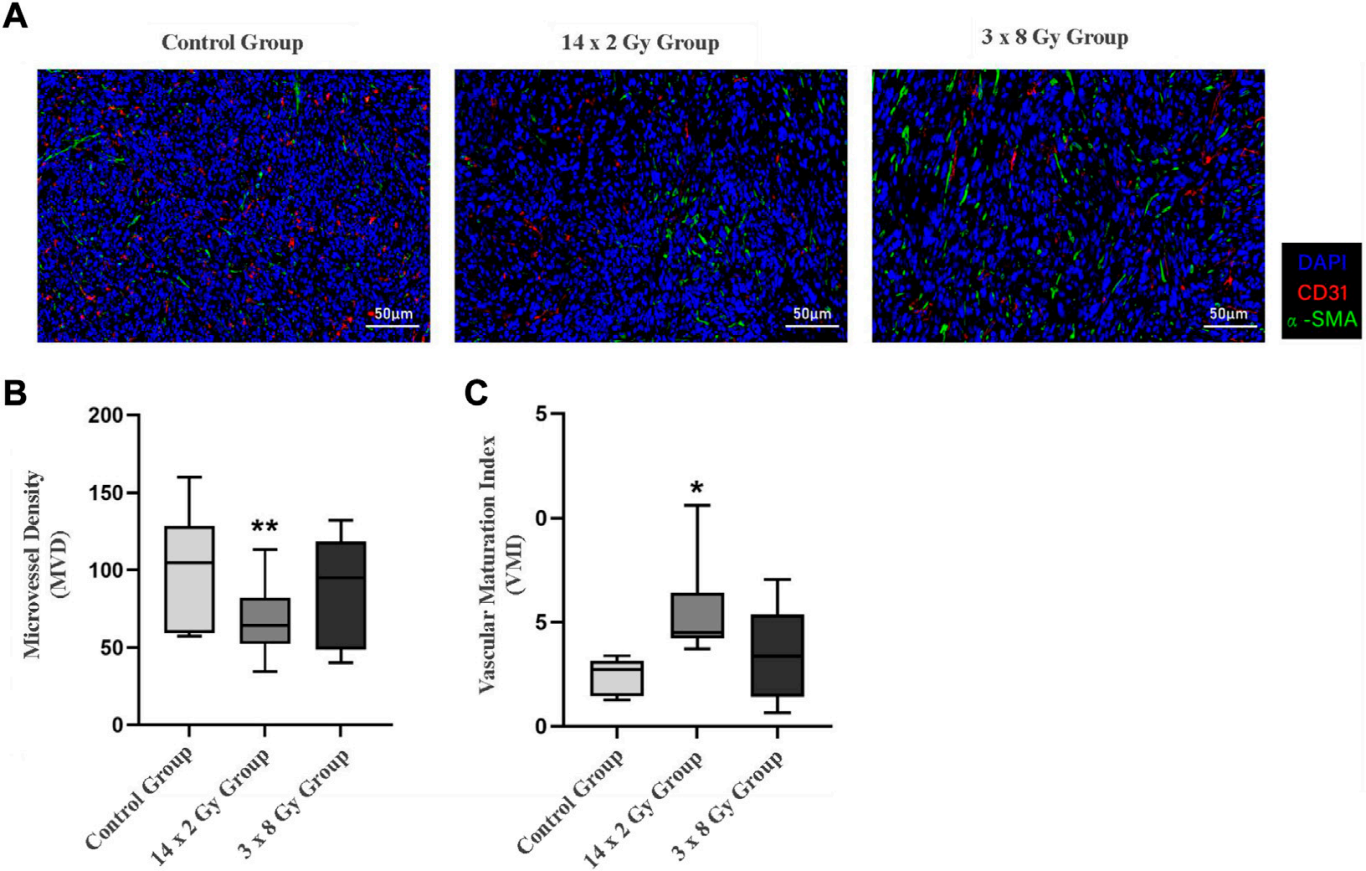
Immunofluorescence analysis of 4T1 breast tumors after irradiation at different doses. **(A)** Shows double-marker immunofluorescence of CD31 (red) and α-SMA (green), and the blue area represents the nucleus. **(B)** Indicates the quantitative expression of microvessel density (MVD) in tumor after radiotherapy. **(C)** Indicates the change of vascular maturity index (VMI) after radiotherapy. Compared with control the control group, *p < 0.05; Compared with control the control group, **p < 0.01.

Tumor sections were stained with CD31-α-SMA to detect tumor microvessels (Figure 5A). The red area represents CD31, the green area representsα-SMA (green), and the blue area represents the nucleus. Microvessel density (MVD) and vascular maturity index (VMI) were used to evaluate the effect of radiotherapy segmentation on tumor angiogenesis. As the tumor grew, compared with the control group, the number of blood vessels decreased, the 14 × 2 Gy group decreased to 37.52%, the 3 × 8 Gy group decreased to 11.54%. However there was a significant difference in MVD between the 14 × 2 Gy group and the control group (*p* < 0.01) (Figure 5B). In addition, compared with the control group, radiotherapy could increase VMI, the 14 × 2 Gy group increased to 40.48%, the 3 × 8 Gy group increased to 19.35%, there was a more significant difference in VMI in the 14 × 2 Gy group (*p* < 0.05) and the increase in 3 × 8 Gy group was not statistically significant (*p* > 0.05) (Figure 5C).

## 4 Discussion and conclusion

A detailed understanding of radiation-induced microvascular changes in tumors is important to maximize the efficacy of radiotherapy. In particular, studying the mechanism of tumor response to a single irradiation is a pressing challenge in radiobiology. A key aspect is the dynamic assessment of the subtle interplay between tumor vascular responses and changes in oxygen status and the contribution of oxygen status changes to the process of driving tumor cell death. In this study, we used high spatiotemporal resolution OA imaging to achieve noninvasive assessment of tumor microvascular morphologic and functional responses. Abundant tumor microvessels can be observed clearly in OA images. The nature of the different (size-dependent) responses of 4T1 breast tumor microvasculature to different doses of radiation as seen from the obtained OA images. Compared with the control group, the vascular density decreased and the number of vascular segments decreased after irradiation. MVD in OA images is the relative value, while that in immunofluorescence images is absolute value. Our results of the overall trend of change of MVD in OA images were roughly consistent with those in immunofluorescence images. The measurement results further showed that the tumor oxygenation level and vascular maturation index increased under 14 × 2 Gy and 3 × 8 Gy irradiation.

Many aspects of vascular biology are affected by radiotherapy and vary according to the radiotherapy protocol. Radiotherapy can cause varying degrees of endothelial cell death and redistribute neoplastic neovascularization by pruning structurally disordered/dysfunctional vessels, but such vascular changes may not translate systematically into long-term effects on the tumor. In a word, Our results indicate that different doses of radiotherapy remodeling tumor blood vessels make tumor vascular structure and function tend to be dynamic balance, improve tumor angiogenesis, and improve the internal reoxidation of tumors. Our findings show the potential of OAI in monitoring the early radiotherapy response of tumor and evaluating the changes of tumor mircovessels and microenvironment. Successful development and application of OAI for tumor imaging could potentially guide the study design of radiotherapy and assess the dose rate, dose fraction and fraction number. Scientific assessment of functional microenvironment changes in response to radiotherapy could also determine the treatment plan in cancer patients.

## Data Availability

The original contributions presented in the study are included in the article/Supplementary Material, further inquiries can be directed to the corresponding authors.
